# Paleogene Radiation of a Plant Pathogenic Mushroom

**DOI:** 10.1371/journal.pone.0028545

**Published:** 2011-12-28

**Authors:** Martin P. A. Coetzee, Paulette Bloomer, Michael J. Wingfield, Brenda D. Wingfield

**Affiliations:** 1 Department of Genetics, Forestry and Agricultural Biotechnology Institute (FABI), University of Pretoria, Pretoria, South Africa; 2 Department of Genetics, Molecular Ecology and Evolution Programme (MEEP), University of Pretoria, Pretoria, South Africa; University of Sydney, Australia

## Abstract

**Background:**

The global movement and speciation of fungal plant pathogens is important, especially because of the economic losses they cause and the ease with which they are able to spread across large areas. Understanding the biogeography and origin of these plant pathogens can provide insights regarding their dispersal and current day distribution. We tested the hypothesis of a Gondwanan origin of the plant pathogenic mushroom genus *Armillaria* and the currently accepted premise that vicariance accounts for the extant distribution of the species.

**Methods:**

The phylogeny of a selection of *Armillaria* species was reconstructed based on Maximum Parsimony (MP), Maximum Likelihood (ML) and Bayesian Inference (BI). A timeline was then placed on the divergence of lineages using a Bayesian relaxed molecular clock approach.

**Results:**

Phylogenetic analyses of sequenced data for three combined nuclear regions provided strong support for three major geographically defined clades: Holarctic, South American-Australasian and African. Molecular dating placed the initial radiation of the genus at 54 million years ago within the Early Paleogene, postdating the tectonic break-up of Gondwana.

**Conclusions:**

The distribution of extant *Armillaria* species is the result of ancient long-distance dispersal rather than vicariance due to continental drift. As these finding are contrary to most prior vicariance hypotheses for fungi, our results highlight the important role of long-distance dispersal in the radiation of fungal pathogens from the Southern Hemisphere.

## Introduction

The biogeography and origin of fungi, in contrast to that of animals and plants, has until relatively recently been a largely neglected area of study. This has been attributed to shortcomings in delimiting species based on morphological species recognition, poor knowledge of the phylogeny of many fungal groups, a poor fossil record, and the view that fungi are able to overcome geographic barriers by virtue of their airborne spores [1]–[4]. However, new insights into dispersal capabilities of fungi and an improved understanding of fungal phylogenetic relationships have shown that fungi provide fascinating subjects for historical biogeography studies [5].

A central debate underlying attempts to explain the biogeography of extant taxa concerns the relative importance of vicariance versus dispersal [6], [7]. The few earlier studies that have dealt with the biogeography of fungi attributed their distribution patterns to vicariance because of their host association and worldwide distribution [8]–[11]. However, applying molecular dating methods, recent studies showed that both vicariance and long-distance dispersal (trans-oceanic and trans-continental) play a role in shaping speciation events and species distributions [3], [5].

Few biogeographical studies on fungi have attempted to use molecular clock dating to determine the time and centre of origin for fungal genera. Rather, these studies have largely been based on data derived from their distribution patterns, phylogenetic relationships, fossil records or mycorrhizal symbiotic associations with trees [12]–[15]. Authors of such studies have, for example, postulated that certain fungi originated in Pangaea and underwent subsequent allopatric speciation after the separation of Laurasia and Gondwana, which was initiated during the Mid Jurassic (180 Million Years Ago [MYA]) (e.g. [13]). In contrast, a more recent Gondwanan center of origin has been proposed for fungi that arose after Pangaea had already separated into two supercontinents [e.g. 14], [15]. Likewise, a Gondwanan origin has been suggested for species of the phytopathogenic fungal genus *Armillaria* (Fr.∶Fr.) Staude [16], [17].

Species of *Armillaria* are generalists that cause root rot in a wide variety of plant hosts. Additionally, they are highly efficient at colonizing new areas owing to their ability to survive not only as pathogens but also as saprobes or necrotrophs on a wide variety of woody plants and plant tissues [18]–[20]. The genus belongs to the group of gilled mushrooms in the Agaricomycetes (including puffballs, bracket fungi, etc.). At least 51 *Armillaria* species are known from tropical, sub-tropical and temperate regions of the world [19]–[22].

An earlier study on the biogeography of *Armillaria* has suggested that this is an ancient genus based on species distribution patterns [23]. The study by Kile et al. [23] included species from the Holarctic (North America, Europe and Far East), sub-Saharan Africa, Indo-Malaysia, the Neotropics (South America, Caribbean and Central America) and Australasia. These authors suggested that the present day distribution of the genus is best explained by adaptive radiation from ancestral forms mediated by the break-up of landmasses.

More recent phylogenetic studies have suggested a Gondwanan origin for *Armillaria* [16], [17]. However, no research has been undertaken that included species from both the Southern and Northern hemispheres and that have employed molecular dating methods to provide a date for the origin of this genus. Piercey-Normore et al. [24] used DNA sequence data from arbitrarily amplified genomic regions for a phylogenetic analysis and dating for North American species. Results from their molecular dating placed the ancestor of these species at 30 MYA, during the late Eocene. A comprehensive account including species from both hemispheres is lacking.

**Figure 1:**
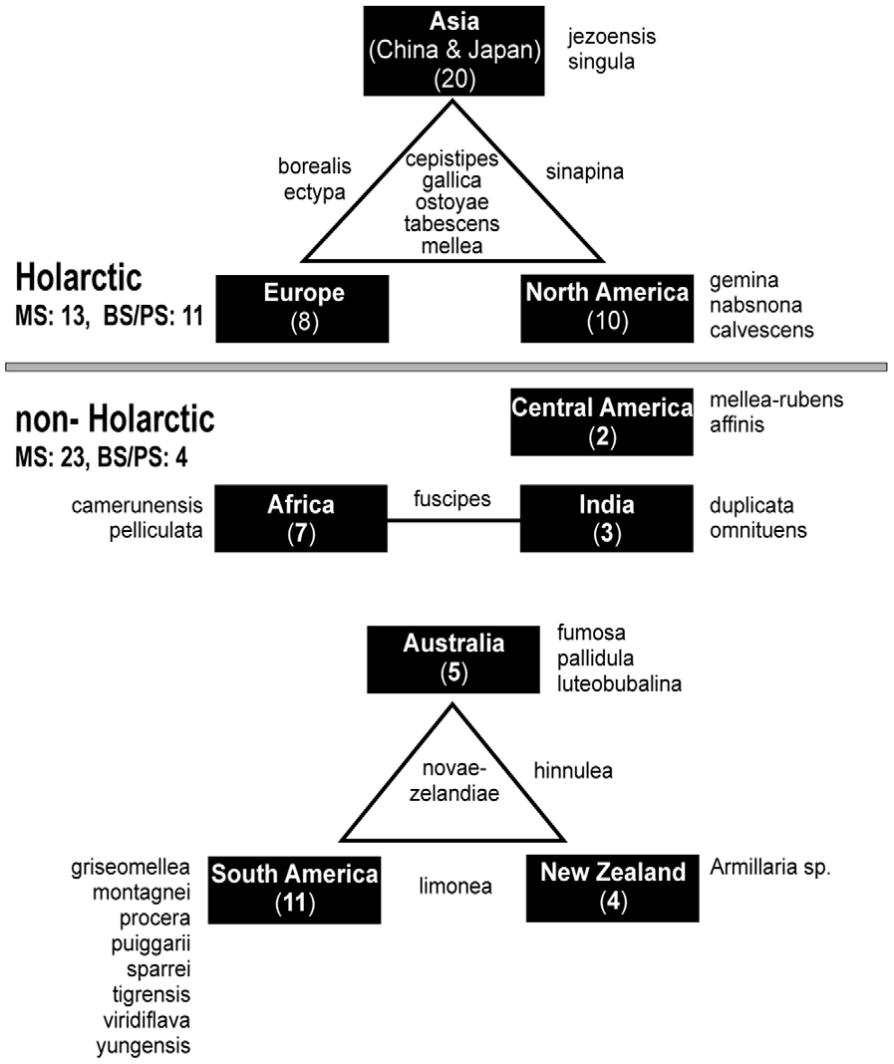
Summary of the global distribution of *Armillaria* spp. Regions are shown in filled rectangles. Species that are unique to a region are listed next to the region name. Species shared by more than one region are indicated in the triangles, those occurring only in two regions are shown between the region names. Numbers in parenthesis indicate the total number of taxa, including morphological (members of a species being congruent in their morphological characteristics [80]), biological (group of organisms that are sexually and reproductively isolated from other groups [81]) and phylogenetic (in the context of *Armillaria* systematics, individuals forming a monophyletic group based on their shared derived characters) species known from each region. (Abbreviations: MS: morphological species, BS: biological species, PS: phylogenetic species).

Neither the manner in which the current distribution of *Armillaria* has occurred nor the date of its radiation is known. The purpose of this study was, therefore, to document the historical biogeography of *Armillaria*. Our working hypothesis was that *Armillaria* originated in Gondwana before the separation of the modern continents. Further, that vicariance events via continental drift have played a major role in mediating speciation. These hypotheses emerged from the known distribution of *Armillaria*, where most species occur exclusively in either the Holarctic or southern non-Holarctic (African, Australian and South American; *sensu* [25]) Floral Kingdoms (**Figure 1**). To test our hypothesis regarding the Gondwanan origin of the genus, we first determined the phylogeny of species of *Armillaria* and their ancestral areas. A temporal dimension was then added to this analysis by estimating the relative age of the genus and the time of divergence among its constituent species.

## Materials and Methods

### *Armillaria* taxon sampling

No specific permits were required for the fungal isolates included in this study. Isolates were collected by ourselves or donated from the private culture collections of colleagues in many other parts of the world. As such no specific permissions were required for using fungal isolates from the regions in this study.

Taxa from the Holarctic were chosen to represent the *A. gallica*, *A. ostoyae*, *A. mellea* and *A. tabescens* “species clusters” described by Korhonen [26] because they cover the range of species across the region. In this study, the *A. gallica* species cluster was represented by *A. gallica, A. cepistipes, A. nabsnona* and Bhutanese Phylogenetics Species 1 (BPS1) (**Table S1**). BPS1 is a new phylogenetic species within *Armillaria* and shown to be closely related to species within the *A. gallica* species cluster by Coetzee et al. [27] and therefore, was included as one of the representatives of this group. The *A. ostoyae* cluster included *A. ostoyae*, *A. borealis* and *A. gemina* (**Table S1**). The *A. tabescens* species cluster constitutes the only known ex-annulated *Armillaria* species, *A. tabescens* and *A. ectypa*; both species were included in the current study (**Table S1**). Coetzee et al. [28] showed that the *A. mellea* species cluster is composed of four geographically seperated clades, referred to as the Asian, European, eastern North American and western North America clades. An *A. mellea* isolate representing each of the clades was thererefore included in this study (**Table S1**). Species and isolates from the Southern Hemisphere Floral Kingdoms are represented mainly, but not exclusively, by those from Africa, Australia and New Zealand (**Table S1**). An earlier phylogenetic study that included isolates from various countries in Africa showed that the African *Armillaria* forms two major lineages, referred to as Clades A and B [29]. These are represented by *A. fuscipes* (Clade A) and *Armillaria* Groups II and III (Clade B) in the current study. The latter taxa are distinct species that await formal description [30]. Species from Australia, New Zealand and Indo-Malaysia included *A. fumosa, A hinnulea, A. limonea, A. luteobubalina, A. pallidula* and *A. novae-zelandiae* as they commonly occur in these regions. With the exception of *A. montagnei* and *A. novae-zelandiae*, cultures for the species reported from South America were unavailable for this study and representation from this region it thus incomplete. Overall, isolates used in this study (**Table S1**) originated from a wide variety of hosts.

### Molecular techniques

DNA extractions, PCR reaction mixtures and PCR conditions followed those outlined by Coetzee et al. [28]. The internally transcribed spacer (ITS) region of the rDNA operon, which includes the ITS 1 region, 5.8S gene and ITS 2 region, was amplified using primers ITS1 and ITS4 [31]. The large subunit (LSU) region of the rDNA operon was amplified with primers LR0R [32] and LR11 [33]. An internal region of the elongation factor one alpha (EF 1-α) gene was amplified using primers EF595F and EF1160R [34] for isolates that were not included in a previous study by Maphosa et al. [35] and for which sequences are not available in GenBank. Sequences were obtained for the ITS and EF 1-α regions in both directions using the same primers employed for PCR. For sequencing the LSU, primers LR0R, LR3R, LR5, LR6, LR7, LR8, LR9, LR11, LR14 and LR17R [32], [33] were used.

### Data matrices for estimating time of divergences

Three data sets were generated for analyses and these were termed the Basidiomycota Matrix, the Ascomycota-Basidiomycota Matrix and the *Armillaria* Matrix. The first two matrices were generated to obtain a secondary calibration date that was subsequently applied to a chronogram obtained from the *Armillaria* Matrix.

The Basidiomycota matrix included LSU sequences for *A. fuscipes*, *A. mellea* and *A. novae-zelandia*, members of the Agaricomycotina and the distantly related Ustilagomycotina [36]. *Armillaria* belongs to the Agaricales, an order that is closely related to the Boletales [37]. Therefore, this data matrix included additional representative from the Agaricales, members of the Boletales as well as representatives from taxon orders which, based on the phylogeny of Matheny et al. [36], represent a range in phylogenetic relatedness to the Agaricales and Boletales. *Puccinia graminis* from the Pucciniomycotina was used as outgroup taxon (**Table S2**).

A recent study by Hibbett and Matheny [38] included RNA polymerase II (RPB2) protein sequences as well as nuclear ribosomal small sub-unit (SSU) DNA sequences from various taxon groups in the Ascomycota and Basidiomycota and also included sequences for *A. mellea*. We, therefore, included these sequences in our Ascomycota-Basidiomycota Matrix (**Table S3**). However, *Flamullina velutipes* and *Xerula radicata*, which are closely related to *Armillaria* and for which sequences were not included in the study of Hibbett et al. [38], were added to the Ascomycota-Basidiomycota Matrix. Trees generated from this data matrix were rooted to *Glomus mosseae* and *Paraglomus occultum*, species that reside in the Glomeromycota, the suggested sister group to the Ascomycota and Basidiomycota [39].

The *Armillaria* Matrix included sequences for the ITS, nearly complete LSU and EF 1-α regions for *Armillaria* spp. (**Table S1**). *Coniophora puteana* (AM293066, AJ583426 and AM293182) and *Serpula lacrymans* (EU162051, AJ440940 and AJ518928) that reside in the Boletales were included as outgroup taxa. Species belonging to the Agaricales were initially included in the *Armillaria* Matrix to serve as outgroup taxa. However, they did not provide sufficient character polarization and, therefore, representatives of the Boletales that are closely related to the Agaricales were selected as outgroup taxa.

### Sequence alignment and selection of nucleotide and protein models of evolution

Sequences were aligned using MAFFT ver. 6 [40]. For this purpose, an iterative refined method (FFT-NS-i) with default parameter settings was used. Data sets can be requested from the corresponding author or obtained online at http://www.fabinet.up.ac.za/people/mpacoetzee.

Nucleotide evolution models and parameter values applicable to the individual partitions were determined with jModelTest ver. 0.1.1 [41] using an Akaike Information Criterion to select the model with the best likelihood. A substitution model for ML analysis was also estimated for the partitions combined in the *Armillaria* Matrix. ProtTest ver. 2.4 [42] was used to select the model of protein evolution that best fits the RPB2 amino acid sequence alignment.

### Phylogenetic analyses of Armillaria

The ITS, LSU and EF 1-α sequences in the *Armillaria* Matrix were tested for combinability using the incongruence length difference test [43], [44], also known as the partition homogeneity test (PHT), implemented in PAUP* ver. 4b10 [45] with 1000 replications.

Maximum parsimony (MP) trees were obtained following a heuristic search with tree bisection reconnection branch swapping and MulTrees effective in PAUP*. Gaps, missing characters, ambiguously aligned and uninformative characters were excluded from the data sets prior to the analysis. Starting trees were obtained via random addition of taxa (100 replicates). MaxTrees was set to auto-increase and zero length branches were collapsed. Weighted parsimonious trees (MPw) were generated by scaling the characters according to their rescaled consistency (RC) index in PAUP*. Subsequent heuristic searches were performed using the same settings as above. Support for tree nodes, for both unweighted and weighted parsimony, was determined using bootstrap analysis (1000 replicates) [46] with the same settings as above, but with starting trees obtained via stepwise addition.

Maximum likelihood (ML) analysis was done using PHYML ver. 2.4.4 [47]. A GTR+G substitution model was applied across the combined dataset for the analysis. Support for tree nodes was obtained using bootstrap analysis with 1000 replicates. Bayesian inference of phylogenies was conducted as explained below where estimation of divergence times is considered.

### Ancestral area reconstruction of Armillaria spp

Ancestral distributions of *Armillaria* species were reconstructed using DIVA ver. 1.1 [48]. For this purpose, a data matrix was generated including the species and their presence or absence in a specific geographical area. The areas were defined as Holarctic (A), Australia (B), New Zealand (C), South America (D), Indo-Malaysia (E) and Africa (F). The topology of the tree generated from the ML analysis was used as the user input tree. The default settings and function values in DIVA were applied for optimization of area reconstruction (see http://www.ebc.uu.se/systzoo/research/diva/manual/dmanual.html, for a detailed description of the utility functions and default values).

### Estimation of divergence times

Estimation of divergence times based on Bayesian inference were applied to the Basidiomycota, Ascomycota-Basidiomycota and *Armillaria* matrices separately, using BEAST ver. 1.5.2 [49]. The settings for BEAST included a relaxed clock with uncorrelated log-normal rate variation across branches with a Yule speciation prior, which assumes a constant speciation rate per lineage. Substitution models, determined with jModelTest or ProtTest (**Table S4**), unique for each data partition were used with values for the parameters unlinked across all partitions. All priors and operators, with the exception of the calibration priors, were the default settings. The Markov Chain Monte Carlo (MCMC) chain was run for 2×10^8^ generations, with sampling frequency set to every 1000 steps. These runs were done four times for all other datasets to ensure convergence. Tracer ver. 1.4.1 (http://tree.bio.ed.ac.uk/software/tracer/) was used to determine convergence, adequate mixing of the MCMC chains, effective sample sizes for model parameters and burnin. TreeAnnotator ver. 1.5.2 [50] was used to calculate divergence dates, 95% highest posterior densities (HPD) for divergence times and posterior probabilities for the nodes, and a consensus tree topology. Trees were viewed in FigTree ver. 1.1.2 (http://tree.bio.ed.ac.uk/software/figtree/).

### Calibration of the relaxed molecular clock

A secondary calibration approach [51] was applied to determine the age of *Armillaria* based on relaxed molecular clock methods. This approach was motivated by the lack of a generally accepted mutation rate for filamentous fungi, as well as the poor fossil record for the Basidiomycota, specifically the absence of fossils belonging to the Physalacriaceae, the fungal family that accommodates *Armillaria*. Furthermore, uncertainty exists regarding the taxonomic placement of some fungal fossils ascribed to the Basidiomycota [38], which complicated calibrations based on fossil records. In addition, because sequences available on GenBank for the Basidiomycota and Ascomycota differed in the genomic regions that were included in the *Armillaria* Matrix, direct calibration of a chronogram generated from a matrix that incorporated taxa from these phyla together with the *Armillaria* species included in this study could not have been done.

The time of divergence between the Agaricales and Boletales was estimated from three analyses, one based on the Basidiomycota Matrix and two using the Ascomycota-Basidiomycota Matrix with different calibration times. Application of these analyses were motivated by the fact that species of Boletales comprised the outgroup for the *Armillaria* Matrix. The time of divergence obtained from these analyses could then be applied to the root height of the chronogram generated from the *Armillaria* Matrix. Also, multiple analyses were done to ascertain whether the different datasets and calibration dates yielded congruent divergence times for the Agaricales and Boletales. This provided a means to gain confidence in the date that was used as the secondary calibration date.

In the first analysis, the approach employed by Matheny et al. [3] was followed by setting the prior distribution for the divergence between *Ustilago* and the Agaricomycotina in the Basidiomycota Matrix to a normal distribution with a mean of 430 million years before present (Myr BP) (SD: 50) [52] (**Table S5**). The second and third analyses employed the Ascomycota-Basidiomycota Matrix. In the second analysis, the molecular clock was calibrated against the time of divergence between the Ascomycota and Basidiomycota. Recently, Lücking et al. [53] provided support for the origin of the Ascomycota between 500–650 million years ago (MYA), but also stated that the origin of the Ascomycota and Basidiomycota is around 500 MYA. Based on their findings, a conservative approach was followed by setting the time since the most recent common ancestor (tMRCA) of the Ascomycota and Basidiomycota to a normal prior with a mean of 575 Myr BP (SD: 26) (**Table S5**). In the third analysis, the prior distribution for the tMRCA of *Ustilago* and the Agaricomycotina was set to a normal distribution with a mean of 430 Myr BP (SD: 50) (**Table S5**).

The analyses outlined above yielded congruent results, placing the time of divergence between the Agaricales and Boletales at 142 MYA (95% Lower HPD: 65 to 95% Upper HPD: 175), 145 MYA (110–201) and 144 MYA (96–206), respectively (**Table S5, Figures S1 and S2**). *Archaeomarasmius leggetti* (dated at *ca.* 95 MYA) is the oldest known fossil belonging to the Agaricales and was suggested to have a close relationship with marasmioid fungi in this order [54]. The older age of this fossil renders the 65 MYA lower range time of divergence between the Agaricales and Boletales obtained from the Basidiomycota Matrix implausible. The time of divergence range obtained from the second analysis was slightly narrower than that estimated from the third analysis. The secondary calibration date generated from the second analysis was therefore used as the root height prior for trees generated from the *Armillaria* Matrix in subsequent analyses. In addition, the tMRCA of *Armillaria* (i.e. node shared by *A. fuscipes*, *A. mellea* and *A. novae-zelandiae*) was determined from the Basidiomycota Matrix to evaluate the time of the *Armillaria* species radiation estimated from the *Armillaria* Matrix.

## Results

### Phylogeny of Armillaria species and determination of their ancestral areas

The PHT indicated some incongruence among the ITS - LSU and EF 1-α sequence partitions (*P* = 0.01) in the *Armillaria* Matrix. Furthermore, phylogenetic trees generated from this matrix that included DNA sequences representing a selection of *Armillaria* species (**Table S1**) suffered from a lack of bootstrap support for some of the deeper nodes. These anomalies could be ascribed to incongruent tree topologies when phylogenetic trees were generated from individual ITS, LSU and EF 1-α sequences. *Armillaria hinnulea* and an undescribed *Armillaria* species from New Zealand were placed in conflicting positions on the individual trees: Both the ITS and LSU sequences placed the two species within the Holarctic group, while they were positioned outside this group based on EF 1-α data. These findings are congruent with previous phylogenetic studies based on EF 1-α [35] and ITS [16], [55] sequences. Preliminary results from a recent study, based on phylogenetic analyses using additional molecular markers, suggest that the conflicting placement of these two taxa is due to incomplete linage sorting (data not shown). Exclusion of the two taxa led to a PHT that failed to show incongruence (*P* = 0.182) and an increase of bootstrap support values for the deeper nodes.

**Figure 2:**
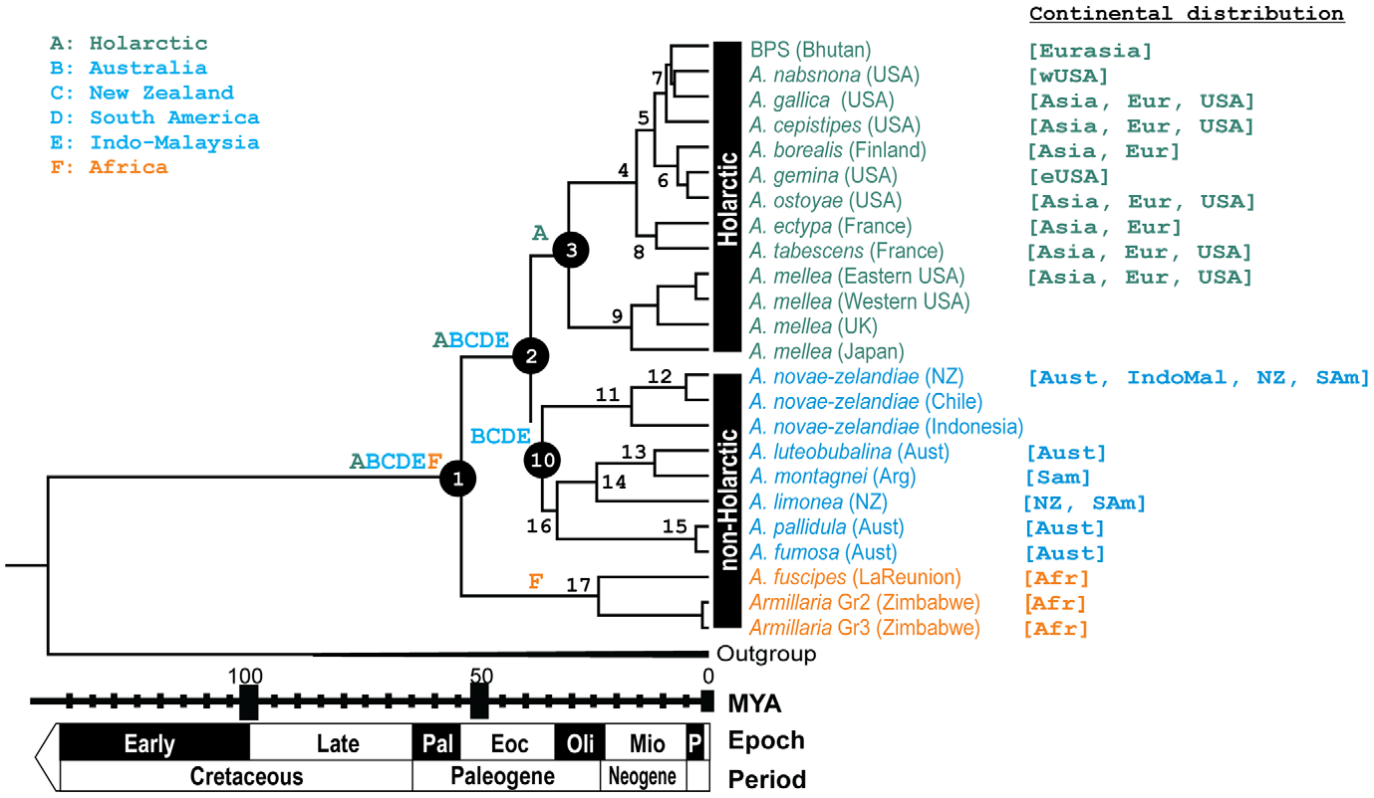
Chronogram generated from combined ITS, LSU and EF-1 α using BEAST (support values for the nodes generated from MP, MPw, ML and BEAST together with their age and 95% Higher Posterior Density values are provided in **Table 1**). Numbers next to the nodes refer to the node numbers in Table 1, the MRCA of extant *Armillaria* spp. as well as the Holarctic lineage and South American-Australasian lineages are encircled. Optimum reconstruction of area distributions are indicated next to the main ancestral nodes on the tree. The origin of the isolates is denoted in brackets next to the species name. The biogeographical distribution for each species is indicated in square brackets. *Coniophora puteana* (AM293066, AJ583426 and AM293182) and *Serpula lacrymans* (EU162051, AJ440940 and AJ518928) that reside in the Boletales were used to root the tree. (Abbreviations: wUSA = western USA, eUSA = eastern USA, Eur = Europe, Aust = Australia, IndoMal = Indo-Malasia, NZ = New Zealand, SAm = South America, Afr = Africa; P = Pliocene; Mio = Miocene; Oli = Oligocene; Eoc = Eocene; Pal = Paleocene.)

Phylogenetic trees generated after the exclusion of *A. hinnulea* and the undescribed *Armillaria* species from New Zealand and using different phylogenetic methods had congruent topologies but with varying statistical support for tree nodes (**Table 1** **,** **Figure 2**). The phylogenetic analyses suggested that the most recent common ancestor (MRCA) of extant *Armillaria* species gave rise to three major lineages: Holarctic, Australasia-South American and African. These clades had high bootstrap support and significant Bayesian posterior probability values (**Table 1** **,** **Figure 2**). Although not well supported by Bayesian analyses, bootstrap analyses based on parsimony and maximum likelihood supported a sister relationship between the Holarctic and Australasia-South American groups, while the deepest split for *Armillaria* separates the African lineage from the remaining species (**Table 1** **,** **Figure 2**).

**Table 1:** Bootstrap and posterior probability values from different phylogenetic methods as well as ages for nodes, heights and confidence intervals on the phylogenetic tree presented in Fig. 2 (major lineages are indicated in bold).

Node	Species/geographical group	MP^1^	MPw^1^	ML^1^	BEAST: Secondary calibration from Ascomycota/Basidiomycota divergence				
					PP^2^	Age	Median node height	95% HPD	
								L^2^	U^2^
1	***Armillaria*** ** MRCA**	100	100	100	1.00	54	54	30	85
2	**Holarctic-South American-Australasian**	99	100	74	0.90	39	41	23	66
3	**Holarctic**	75	95	80	0.99	31	33	19	56
4	*A. ostoyae* group	67	86	89	0.99	7	8	3	15
5	*A. gallica* group	74	69	90	1.00	9	8	3	15
6	*A. ostoyae A. gallica*	77	94	87	1.00	12	12	5	22
7	*A. tabescens, A. ectypa*	98	100	100	1.00	11	14	6	25
8	*A. tabescens, A. ectypa, A. ostoyae A. gallica*	87	97	92	1.00	16	23	12	38
9	*A. mellea* group	100	100	100	1.00	17	18	9	33
10	**South American – Australasian**	61	69	75	0.98	36	33	18	55
11	*A. novae-zelandia* (Aust,SAm^1^)	100	100	100	1.00	5	3	1	8
12	*A. novae-zelandia* (Aust,SAm, Indonesia)	100	100	100	1.00	17	15	7	29
13	*A. fumosa, A. pallidula*	100	100	100	1.00	3	3	1	8
14	*A. luteobubalina*	100	100	100	1.00	12	9	3	18
15	*A. luteobabalina, A. limonea*	65	96	66	1.00	24	21	10	37
16	*A. luteobabalina, A. limonea, A. fumosa,A. pallidula*	63	84	82	1.00	33	28	15	46
17	African	100	100	100	1.00	24	18	8	34

1 Abbreviations: Aus: Australia, NZ: New Zealand, SAm: South America, MP: maximum parsimony, MPw: weighted maximum parsimony, ML: maximum likelihood.

2 PP: posterior probability; L: 95% Lower Highest Posterior Density, U: 95% Upper Highest Posterior Density.

Results of ancestral area analyses indicated that two major isolation events occurred during the evolution of the extant *Armillaria* (**Figure 2**). The African lineage became isolated from the ancestral *Armillaria* lineage occurring in the remaining regions. Subsequently, the Holarctic and Australasia-South American lineages diverged, followed by independent cladogenesis in the respective regions.

### Age and radiation of Armillaria

The age of the ancestor of *Armillaria* spp., *Xerula radicata* and *Flammulina velutipes* was estimated at 70 million years (MY) (95% Lower HPD: 37 to 95% Upper HPD: 75) from the Basidiomycota Matrix and 59 (32–90) MY from the Ascomycota-Basidiomycota Matrix (**Figures S1 and S2**). The tMRCA of *A. fuscipes, A. mellea* and *A. novae-zelandia* was estimated at 54 MY (22–75) from the Basidiomycota Matrix (**Figures S1 and S2**). When the date of divergence between the Agaricales and Boletales was used as a constraint for the height of the tree generated from the *Armillaria* Matrix in dating analysis, the tMRCA of *Armillaria* was determined to be 54 MY (30–85) (**Table 1**, node 1: **Figure 2**). The age of the MRCA of the Holarctic – South American - Australasian taxa was estimated at 39 MY (23–66) (node 2: **Figure 2**). The radiation of the Holarctic taxa (node 3: **Figure 2**) was estimated at 31 MYA (19–56), the South American-Australasian taxa (node 10: **Figure 2**) at 36 MYA (18–55) and the African taxa (node 17: **Figure 2**) at 24 MYA (8–34) MYA (**Table 1**).

## Discussion

### The MRCA postdates the break-up of Gondwana and supports long-distance dispersal in the distribution of *Armillaria*

Results of this study support the radiation of *Armillaria* from the Southern Hemisphere, well after the fragmentation of Gondwana. This requires long-distance dispersal rather than vicariance as the explanation for current distributions. It, therefore, does not support our initial hypothesis that *Armillaria* originated in Gondwana and that subsequent radiation is the result of vicariance. Dating analyses placed the ancestor of *Armillaria, X. radicata* and *F. veluptipes* at 70 MYA from the Basidiomycota Matrix and 59 MYA from the Ascomycota-Basidiomycota Matrix. The MRCA of extant *Armillaria* spp. was also dated to 54 MYA during the Early Eocene, regardless of the data matrix and calibration date.

Fragmentation of Gondwana began approximately 150 MYA [56], long before *Armillaria* appeared. Additionally, the estimated age of the MRCA for the Holarctic - South American - Australasian taxa at 39 MY is long after Africa separated from southern South America via the opening of the Weddell Sea (*ca.* 160 MYA) [56] and the opening of the South Atlantic Ocean (*ca.* 135 MYA) [57]. It is also placed after Tasmantis, including New Zealand and New Caledonia, began to drift from Gondwana *ca.* 80 MYA [56], [57], with the spread of the Tasman sea persisting until *ca.* 55.5 MYA [56]. The MRCA of the South American - Australasian taxa was similarly placed at ages too young to involve vicariance at *ca.* 36 and 42 MYA for two analyses, respectively.

Analyses in this study indicate that the African *Armillaria* lineage had been separated from the South American lineage for an extended period of time subsequent to initial divergence from the MRCA (**Figure 2**). This is supported by the significantly longer ML branch that separated them from the other lineages. Since Africa had already separated from the remainder of Gondwana by the time the MRCA of the African *Armillaria* lineage had diverged, two alternative hypotheses support the distribution pattern of the African lineage. One is that the African colonisation is the product of ancient long-distance dispersal from South America. Although not supported by the ancestral area analysis, a possible alternative is that the African lineage may have given rise to the temperate austral and north temperate lineages and subsequently spread from Africa to South America. Given the lack of specimens from many parts of the African continent, these two hypotheses cannot be tested. Nevertheless, the results of the phylogenetic analyses suggest a non-Holarctic origin for *Armillaria*.

The radiation of the South American-Australasian *Armillaria* taxa from the MRCA was placed at a time when the separation between East Antarctica and Australia was near completion [56]. Ancient stepping-stone and long-distance dispersal of the ancestral populations between South America and Australia thus seem to be the most reasonable explanation for their current distribution. The occurrence of recently diverged species such as *A. novae-zelandiae* in Australia, New Zealand and South America, distantly separated by oceans, can be accounted for in terms of more recent trans-oceanic long-distance dispersal, similar to that described for plants [57], [58] and other fungi [59], [60].

Two alternative hypotheses, both involving long-distance trans-continental dispersal, incremental dispersal over land and vicariance, are available to explain the current distributions of the Holarctic *Armillaria* taxa. One hypothesis is that the Holarctic taxa dispersal route was overland from South America to North America and then to Eurasia via the Bering land bridge (100–3.5 MYA), when continuous forests existed between North America and northern Asia [61]. Another possibility is that dispersal occurred from the southern continents to Asia, with subsequent spread from Asia overland to Europe and via the Bering land bridge to Northern America. In both scenarios, species would became separated and subsequently diversified through vicariance with the final sundering of the Bering land bridge 5.5–5.4 MYA [62]. While these alternative hypotheses are difficult to resolve, the large number of *Armillaria* species in Asia (**Figure 1**) lends support to the latter hypothesis.

The distribution and global population structure of, for example, *A. mellea* provides credence to the scenario described above. This fungus occurs throughout the Holarctic, but in North America is restricted mainly to the eastern and western coasts with a limited occurrence in central United States [63], southeastern Canada [64] and northeastern Mexico [65]. Results of phylogenetic analyses conducted by Coetzee et al. [28], Maphosa et al. [35] and in the current study, showed that isolates from Europe, eastern Asia, eastern North America and western North America are separated into distinct clades reflecting their geographical origin. It was suggested by Coetzee et al. [28] that these clades represent populations that are in the process of speciation. A recent population genetics study revealed genetic divergence between the eastern and western U.S.A. populations of *A. mellea* [66]. Shared loci (two loci having different allele size and three with the same allele size) were discovered between the two populations by Baumgartner et al. [66]. It was proposed that, due to the vast distance between the two populations and restrictions to gene flow by physical barriers such as Great Plains and Rocky Mountains, the loci shared by the eastern and western populations is the result of a common ancestral origin, rather than ongoing gene flow [66]. Dating in the present study placed the tMRCA of *A. mellea* at 17 (33 – 9) MYA, which would have allowed migration of ancient populations across the Bering land bridge and over land to attain a trans-Holarctic distribution. Vicariance events such as the opening of Bering Strait and the development of belts of arid climate at the centre of North America and Eurasia [67] would subsequently have acted to restrict gene flow leading to genetic diversification and speciation.

Two complementary mechanisms are thus required to account for the distribution patterns observed for *Armillaria* species: Long-distance trans-continental dispersal and incremental dispersal over land. However, if long-distance dispersal was commonplace among *Armillaria* spp., a cosmopolitan distribution for most members of the genus would be expected. This is not the case. There is a clear disjunction between Holarctic and non-Holarctic species. Apart from *Armillaria* species that have probably been introduced into new areas by humans [68], [69], we are not aware of any species that has a global distribution. Hence, while this study provides evidence of long-distance dispersal of *Armillaria*, factors such as adaptation to local climate and environment restrict worldwide spread of taxa.

### Dispersal mechanisms responsible for the distribution of Armillaria

The dispersal process hypothesized here assumes that two major mechanisms were involved in shaping the geographical distribution of extant *Armillaria* species. The first is long-distance trans-continental dispersal that would account for the recent dispersal of extant species between Australasia and South America after these landmasses became separated. The second is incremental dispersal, which involves the gradual spread of a population over vast land areas. This would account for the earlier radiation of ancestral taxa from South America via Antarctica to Australia or *vice versa*, as well as for the radiation of taxa from the Holarctic.

Long-distance dispersal of *Armillaria* spp. prior to anthropogenic influences could potentially entail trans-oceanic dissemination of basidiospores by wind, followed by subsequent spread of the fungus to new areas after successful establishment. Although it is known that basidiospores from the majority of mushroom species do not move great distances [70]–[72], it is possible that fungal spores could disperse by high-altitude winds. Evidence of this phenomenon is provided, for example, by the trans-Atlantic migration of mushroom spores on the North Atlantic air current [73], and the long-distance dispersal of the rust fungus *Puccinia graminis* f.sp. *tritici* from southern Africa to Australia [74]. In a recent study, based on molecular dating and nested clade analysis, it was postulated that trans-oceanic spore dispersal by wind may have facilitated the spread of populations of the root pathogen *Ganoderma australe*, a species with similar life-history to that of *Armillaria*, to continents across the Southern Hemisphere [60].

Unlike long-distance trans-continental dispersal, the incremental dispersal of *Armillaria* species over land need not rely only on spore dispersal. If suitable nutrient sources are available, a population could also spread into a new territory through the growth of “shoe-lace” growth forms, known as rhizomorphs, or vegetative mycelium. Rhizomorphs are specialised morphological adaptations that enable the fungus to search for new hosts by growing out from their food base into new environments that initially do not need to support their growth [75]. Species of root rot fungi, including *Armillaria*, are able to spread from one host to another by growing from an infected host via rhizomorphs or vegetative mycelium to an uninfected host when their roots are in contact. The boreal forests that existed across North America and Europe were sufficiently homogeneous and ubiquitous that a fungus such as *Armillaria* would have had no obvious host barriers to its movement. In addition, it has been estimated that a colony of *A. bulbosa* [ = *A. gallica*] grows at approximately 0.2 m per year [76]. Over a few million years *Armillaria* could theoretically have moved thousands of kilometres. Thus, the movement and spread of *Armillaria* species across the Northern Hemisphere is logically explained by incremental dispersal with subsequent isolation from the Southern Hemisphere.

### Concluding remarks

The work presented in this study adds to our understanding of the evolutionary history of fungi. Results emphasize the complexities encountered when inferring the mechanisms responsible for the distribution of species from their current distribution patterns, their phylogenies, or a combination of these data. We have presented an example of a fungal genus with clear Gondwanan distribution patterns. A *prima facie* assumption, based on distribution patterns, would thus be that Southern Hemisphere member species radiated by means of vicariance. However, this is contradicted by dating of the divergence times for the major extant *Armillaria* taxa.

DNA-based dating placed the radiation of extant *Armillaria* spp. from the MRCA in the Early Eocene, *after* the breakup of Gondwana began. Moreover, subsequent separation of continents in the Southern Hemisphere (e.g. New Zealand and South America) generally predates the divergence of *Armillaria* spp. found on those continents. These species could, therefore, only have achieved their current distributions through long-distance dispersal events. The results of this study add to a growing body of evidence, gathered from several disciplines cf. [77]–[79], that vicariance alone is not sufficient to explain the geographical radiation and speciation events of taxa with a Gondwanan distribution.

## Supporting Information

Figure S1 **Chronogram generated using LSU sequence data to determine a secondary calibration date for the divergence between the Agaricales and Boletales from the Basidiomycota matrix.** Asterisks indicate nodes with PP<0.95.
(TIF)

Figure S2 **Chronogram generated using DNA sequence data from the SSU and LSU genes as well as RPB2 amino acid sequence data to determine a secondary calibration date for the divergence between the Agaricales and Boletales from the Ascomycota - Basidiomycota matrix.** Asterisks indicate nodes with PP<0.95.
(TIF)

Table S1 **Culture numbers and GenBank accessions of** ***Armillaria*** **strains used in this study.**
(DOC)

Table S2 **Data sets and calibration dates used to determine the time of divergence between the Boletales and Agaricales, and the tMRCA of** ***A. fuscipes, A. mellea*** **and** ***A. novae-zealandiae*** **, as well as their node ages, heights and confidence intervals.**
(DOC)

Table S3 **List of species included in the Basidiomycota matrix and their GenBank accession numbers.**
(DOC)

Table S4 **GenBank accession numbers and genome project sources for DNA and amino acid sequence data used in the Ascomycota – Basidiomycota data.**
(DOC)

Table S5 **Substitution models determined from jModelTest and ProtTest during the study.**
(DOC)
