# Supplementary figures and images for: Paleogene Radiation of a Plant Pathogenic Mushroom
Source: PLoS One. 2011 Dec 28;6(12):e28545. doi: 10.1371/journal.pone.0028545 (PMC3247210; doi:10.1371/journal.pone.0028545)

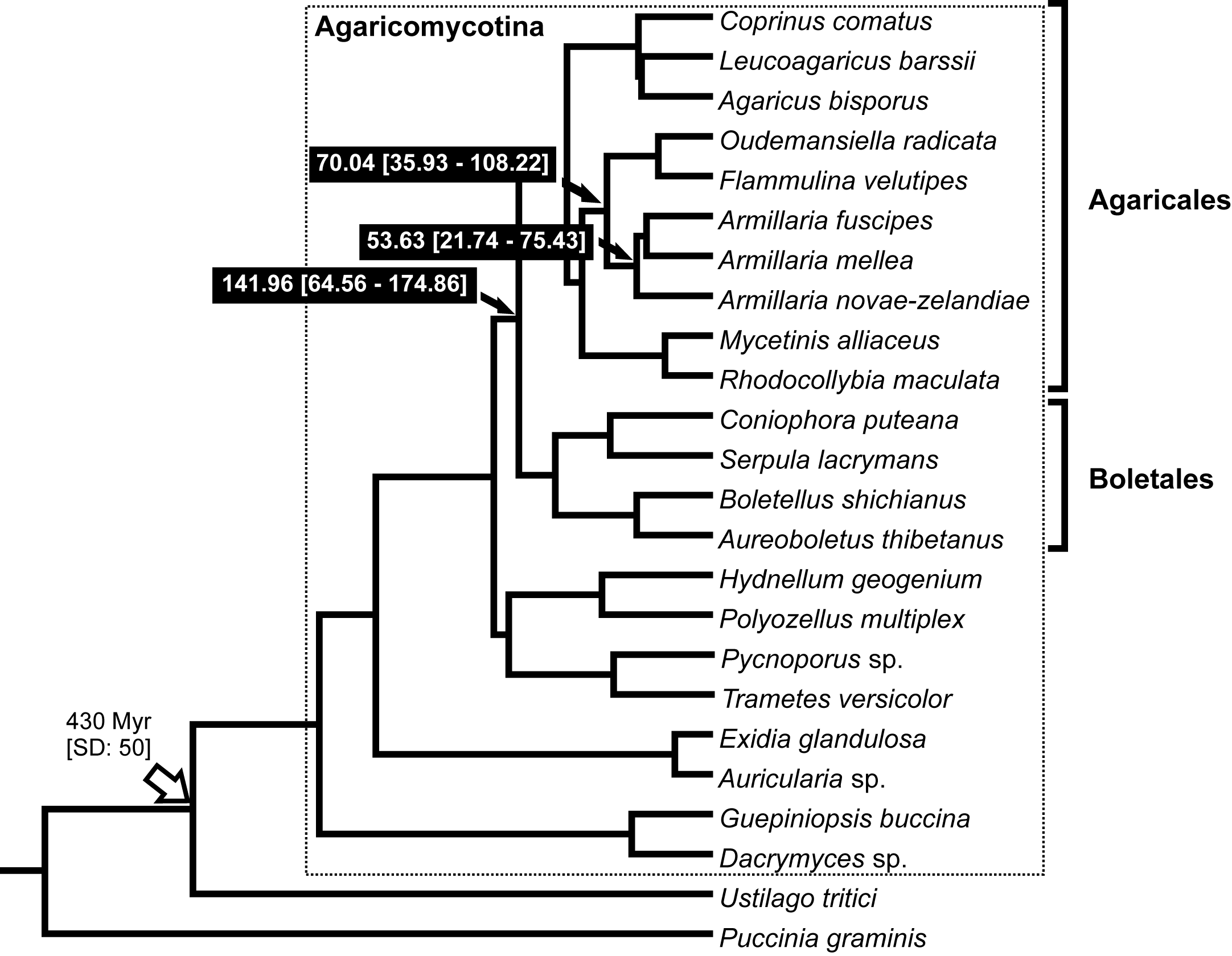

Supplement: Figure S1 — Chronogram generated using LSU sequence data to determine a secondary calibration date for the divergence between the Agaricales and Boletales from the Basidiomycota matrix. Asterisks indicate nodes with PP<0.95. (TIF) [file pone.0028545.s001.tif]

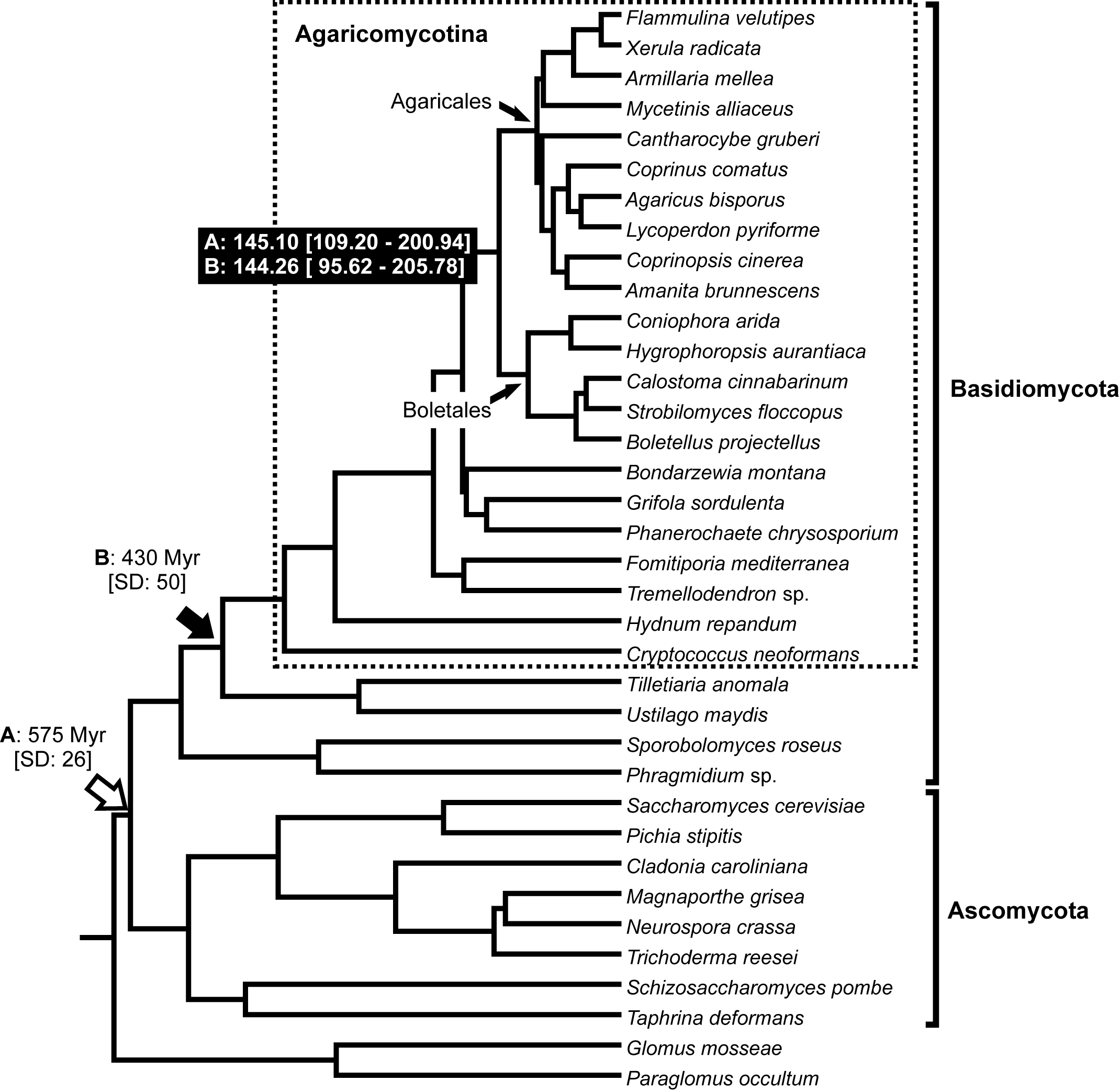

Supplement: Figure S2 — Chronogram generated using DNA sequence data from the SSU and LSU genes as well as RPB2 amino acid sequence data to determine a secondary calibration date for the divergence between the Agaricales and Boletales from the Ascomycota - Basidiomycota matrix. Asterisks indicate nodes with PP<0.95. (TIF) [file pone.0028545.s002.tif]
