# Supplementary material for: Paleogene Radiation of a Plant Pathogenic Mushroom
Source: PLoS One. 2011 Dec 28;6(12):e28545. doi: 10.1371/journal.pone.0028545 (PMC3247210; doi:10.1371/journal.pone.0028545)
Supplement: Table S1 — Culture numbers and GenBank accessions of Armillaria strains used in this study. (DOC) [file pone.0028545.s003.doc]

| **Table S1.** Culture numbers and GenBank accessions of *Armillaria* strains used in this study | | | | | | |
| --- | --- | --- | --- | --- | --- | --- |
| **Species** | **ITS** | | **EF 1-alpha** | | **LSU** | |
|  | Strain number^1^ | GenBank accession number | Strain number | GenBank accession number | Strain number | GenBank accession number |
| **Holarctic** |  |  |  |  |  |  |
| *A. borealis* | UASWS0031 | AY848942 | CMW3172 | DQ435623 | CMW3172 | DQ338540 |
| *A. cepistipes* | W113 | AY213583 | CMW6909 | DQ435631 | CMW6909 | DQ338563 |
| *A. ectypa* | E1 | U54819 | CMW15693 | FJ875698 | CMW15693 | DQ338547 |
| *A. gallica* | CMW7204 | AY190248 | CMW6901 | DQ435628 | CMW6902 | DQ338541 |
| *A. gemina* | ST8 | AY213555 | CMW6888 | DQ435626 | CMW6888 | DQ338543 |
| *A. mellea* (Asia) | B917 | AF163593 | CMW3961 | DQ435638 | CMW3961 | FJ875693 |
| *A. mellea* (Europe) | CMW11265 | FJ875692 | CMW11265 | DQ435637 | CMW11265 | FJ875694 |
| *A. mellea* (eastern USA) | CMW4605 | AF163587 | CMW4605 | DQ435633 | CMW4605 | DQ338545 |
| *A. mellea* (western USA) | B927 | AF163595 | CMW3964 | DQ435634 | CMW3964 | FJ875695 |
| *A. nabsnona* | C21 | AY213572 | CMW3609 | DQ435630 | CMW6905 | DQ338542 |
| *A. ostoyae* | F14310 | AY228342 | CMW3162 | DQ435625 | CMW3162 | DQ338549 |
| *A. tabescens* | ss511 | AY695409 | CMW3165 | DQ435642 | CMW3165 | DQ338546 |
| Bhutanese Phylogenetic Species 1 (BPS1) | CMW10581 | AY554329 | CMW10581 | FJ875699 | CMW10581 | DQ338548 |
| **non-Holarctic** |  |  |  |  |  |  |
| *A. fumosa* | CMW4955 | AF329918 | CMW4955 | DQ435646 | CMW4955 | DQ338552 |
| *A. fuscipes* | CMW4953 | AY882974 | CMW4953 | DQ435622 | CMW4953 | DQ338556 |
| *A. hinnulea* | CMW4990 | AF329905 | CMW4980 | DQ435648 | CMW4980 | DQ338555 |
| *A. limonea* | CMW4680 | AF329930 | CMW4680 | DQ435655 | CMW4680 | DQ338560 |
| *A. luteobubalina* | CMW4977 | AF329912 | CMW4977 | DQ435657 | CMW4977 | DQ338559 |
| *A. montagnei* | CMW5446 | AF448422 | CMW5446 | DQ338562 | CMW5446 | DQ435650 |
| *A. novae-zelandiae* (NZ) | CMW4722 | AF329926 | CMW4722 | DQ435652 | CMW4722 | DQ338551 |
| *A. novae-zelandiae* (Chile) | CMW5448 | AF448417 | CMW5448 | DQ435653 | CMW5448 | DQ338554 |
| *A. novae-zelandiae* (Indonesia / Malaysia) | CMW4143 | AF448421 | CMW4143 | DQ435654 | CMW3951 | DQ338553 |
| *A. pallidula* | CMW4968 | AF329915 | CMW4971 | DQ435647 | CMW4968 | DQ338553 |
| Zimbabwean Group 2 | CMW4456 | AY882984 | CMW4456 | DQ435617 | CMW4456 | DQ338557 |
| Zimbabwean Group 3 | CMW10115 | AY882983 | CMW10115 | DQ435619 | CMW10115 | FJ875696 |
| New Zealand *Armillaria* sp. | CMW4994 | AF329932 | CMW4994 | DQ435643 | CMW4994 | FJ875697 |
