# Supplementary material for: Paleogene Radiation of a Plant Pathogenic Mushroom
Source: PLoS One. 2011 Dec 28;6(12):e28545. doi: 10.1371/journal.pone.0028545 (PMC3247210; doi:10.1371/journal.pone.0028545)
Supplement: Table S2 — Data sets and calibration dates used to determine the time of divergence between the Boletales and Agaricales, and the tMRCA of A. fuscipes, A. mellea and A. novae-zealandiae , as well as their node ages, heights and confidence intervals. (DOC) [file pone.0028545.s004.doc]

**Table S2** List of species included in the Basidiomycota matrix and their GenBank accession numbers

| **Species** | **Genbank accession number** |
| --- | --- |
| *Agaricus bisporus* | AY635775 |
| *Aureoboletus thibetanus* | AY700189 |
| *Auricularia sp.* | AY634277 |
| *Boletellus shichianus* | AY647211 |
| *Coniophora puteana* | AJ583426 |
| *Coprinus comatus* | AY635772 |
| *Dacrymyces* sp. | AY691892 |
| *Exidia glandulosa* | AY293179 |
| *Flammulina velutipes* | AY639883 |
| *Guepiniopsis buccina* | AY745711 |
| *Hydnellum geogenium* | AY631900 |
| *Leucoagaricus barssii* | DQ911601 |
| *Mycetinis alliaceus*  = (*Marasmius alliaceus*) | AY635776 |
| *Xerula radicata*  = (*Oudemansiella radicata*) | AY645051 |
| *Polyozellus multiplex* | AY634275 |
| *Puccinia graminis* | AF522177 |
| *Pycnoporus* sp. | AY684160 |
| *Rhodocollybia maculata* | AY639880 |
| *Serpula lacrymans* | AJ440940 |
| *Trametes versicolor* | AY684159 |
| *Ustilago tritici* | DQ094784 |
