# Supplementary material for: Paleogene Radiation of a Plant Pathogenic Mushroom
Source: PLoS One. 2011 Dec 28;6(12):e28545. doi: 10.1371/journal.pone.0028545 (PMC3247210; doi:10.1371/journal.pone.0028545)
Supplement: Table S3 — List of species included in the Basidiomycota matrix and their GenBank accession numbers. (DOC) [file pone.0028545.s005.doc]

**Table S3** GenBank accession numbers and genome project sources for DNA and amino acid sequence data used in the Ascomycota – Basidiomycota data matrix (Modified from Hibbett & Matheny, 2009).

| **Species** | **Genome project sources and GenBank accession numbers** | | |
| --- | --- | --- | --- |
|  | **RPB2** | **SSU** | **LSU** |
| **Agaricales** |  |  |  |
| *Agaricus bisporus* | AAF19057 | AY787216 | AY635775 |
| *Amanita brunnescens* | AY780936 | AY787216 | AY631902 |
| *Armillaria mellea* | AY780938 | AY787217 | AY700194 |
| *Cantharocybe gruberi* | DQ385879 | DQ234546 | DQ234540 |
| *Coprinopsis cinerea* | XP_001829140 | M92991 | AF041494 |
| *Coprinus comatus* | AY780934 | AY665772 | AY635772 |
| *Flammulina velutipes* | AAV53361 | AY665781 | AY639883 |
| *Lycoperdon pyriforme* | AY218495 | AF026619 | AF287873 |
| *Mycetinis alliaceus*  = (*Marasmius alliaceus*) | AY786060 | AY787214 | AY635776 |
| *Xerula radicata*  = (*Oudemansiella radicata*) | AAV53373 | AY654884 | AY645051 |
| **Agaricomycetes** |  |  |  |
| *Fomitiporia mediterranea* | AY803748 | AY662664 | AY684157 |
| *Grifola sordulenta* | AY786058 | AY665780 | AY645050 |
| *Hydnum repandum* | AAS67513 | AF026641 | AY700199 |
| *Phanaerochaete chrysosporium* | JGI^1^ | U59084 | AF287883 |
| *Tremellodendron* sp. | DQ408132 | AY766081 | AY745701 |
| **Boletales** |  |  |  |
| *Boletellus projectellus* | AY787218 | AY662660 | AY684158 |
| *Calostoma cinnabarinum* | AY780939 | AY665773 | AY645054 |
| *Coniophora arida* | DQ366282 | AY293123 | AF098375 |
| *Hygrophoropsis aurantiaca* | AY786059 | AY662663 | AY684156 |
| *Strobilomyces floccopus* | AY786065 | AY662661 | AY684155 |
| **Pucciniomycetes** |  |  |  |
| *Phragmidium* sp. | AY485630 | EF014363 | AJ715522 |
| *Sporobolomyces roseus* | JG^2^ | DQ832235 | DQ832234 |
| *Bondarzewia montana* | AY218474 | AF026575 | DQ234539 |
| **Tremellomycetes** |  |  |  |
| *Cryptococcus neoformans* | AY485620 | X60183 | L14068 |
| **Ustilaginomycetes** |  |  |  |
| *Tilletiaria anomala* | AY803750 | AY803752 | AY745715 |

Table S4 (continued)

| **Species** | **Genome project sources and GenBank accession numbers** | | |
| --- | --- | --- | --- |
|  | **RPB2** | **SSU** | **LSU** |
| *Ustilago maydis* | EAK83484 | X62396 | AF453938 |
| **Ascomycota** |  |  |  |
| *Cladonia caroliniana* | AY584684 | AY584664 | AY584640 |
| *Magnaporthe grisea* | XP362269 | AB026819 | AB026819 |
| *Neurospora crassa* | AF107789 | X04971 | AF286411 |
| *Pichia stipitis* | JGI^3^ | AB053235^4^ | U75728 |
| *Saccharomyces cerevisiae* | M15693 | J01353 | J01355 |
| *Schizosaccharomyces pombe* | D13337 | X54866 | Z19578 |
| *Taphrina deformans* | AY485633 | U00971 | DQ470973 |
| *Trichoderma reesei* | JGI^5^ | AF548102 | AY544649 |
| **Outgroup** |  |  |  |
| *Glomus mosseae* | EF014400 | AY635833 | DQ273793 |
| *Paraglomus occultum* | DQ826038 | DQ322629 | DQ273827 |

^1^http://genome.jgi-psf.org/Phchr1/Phchr1.home.html

^2^http://genome.jgi-psf.org/Sporo1/Sporo1.home.html

^3^http://genome.jgi-psf.org/Picst3/Picst3.home.html

^4^As *Pichia nakasei*

^5^http://genome.jgi-psf.org/Trire2/Trire2.home.html

**References**

Hibbett, D. & Matheny, P.B. (2009) The relative ages of ectomycorrhizal mushrooms and their plant hosts estimated using Bayesian relaxed molecular clock analyses. *BMC Biology*, **7**, 13.
