# Supplementary material for: Paleogene Radiation of a Plant Pathogenic Mushroom
Source: PLoS One. 2011 Dec 28;6(12):e28545. doi: 10.1371/journal.pone.0028545 (PMC3247210; doi:10.1371/journal.pone.0028545)
Supplement: Table S4 — GenBank accession numbers and genome project sources for DNA and amino acid sequence data used in the Ascomycota – Basidiomycota data. (DOC) [file pone.0028545.s006.doc]

**Table S4:** Substitution models determined from jModelTest and ProtTest during the study.

| **Data matrix** | **Gene** | **Model** |
| --- | --- | --- |
| Basidiomycota | LSU | GTR+I+G |
| Ascomycota – Basidiomycota | SSU | TRN+G |
| Ascomycota – Basidiomycota | LSU | GTR+I+G |
| Ascomycota – Basidiomycota | RPB2 | LG+I+F |
| *Armillaria* | ITS | TVM+I+G* |
| *Armillaria* | LSU | TIM+I+G* |
| *Armillaria* | EF-1α | TIMef+I+G* |

* The invariable sites parameter (I) was excluded from the maximum likelihood and BEAST analyses as this parameter greatly deflated the maximum likelihood bootstrap and poster probability values.
