# Supplementary material for: Paleogene Radiation of a Plant Pathogenic Mushroom
Source: PLoS One. 2011 Dec 28;6(12):e28545. doi: 10.1371/journal.pone.0028545 (PMC3247210; doi:10.1371/journal.pone.0028545)
Supplement: Table S5 — Substitution models determined from jModelTest and ProtTest during the study. (DOC) [file pone.0028545.s007.doc]

| **Table S5.** Data sets and calibration dates used to determine the time of divergence between the Boletales and Agaricales, and the tMRCA of *A. fuscipes, A. mellea* and *A. novae-zealandiae,* as well as their node ages, heights and confidence intervals*.* | | | | | | |
| --- | --- | --- | --- | --- | --- | --- |
| **Dataset** | **Calibration** | **Age** | **Median node height** | **95% HDP** | | |
|  |  |  |  | L | | U |
| **Time of divergence between Boletales / Agaricales** | | | | | | |
| Basidiomycota | 430 (STD: 50) MYA - Divergence of Ustilago and Agaricomycotina | 142 | 150 | 87 | 223 | |
| Ascomycota / Basidiomycota | 575 (STD: 13) MYA - Divergence of Ascomycotina and Basidiomycotina | 145 | 151 | 109 | 201 | |
| Ascomycota / Basidiomycota | 430 (STD: 50) MYA - Divergence of Ustilago and Agaricomycotina | 144 | 150 | 96 | 206 | |
| **tMRCA of the *Armillaria* ancestor** | | | | | | |
| Basidiomycota | 430 (STD: 50) MYA - Divergence of Ustilago and Agaricomycotina | 54 | 45 | 22 | 75 | |
|  |  |  |  |  |  | |
